# Posterior hybrid surgery for atlantoaxial dislocation coexisting with multilevel cervical spondylotic myelopathy

**DOI:** 10.3389/fsurg.2023.1164298

**Published:** 2023-06-02

**Authors:** Yan Sun, Haoning Ma, Zhihai Zhang, Mingsheng Tan

**Affiliations:** ^1^ Department of Orthopaedic Surgery, Guang’an Men Hospital, China Academy of Chinese Medical Sciences, Beijing, China; ^2^ College of Basic Medicine, Beijing University of Chinese Medicine, Beijing, China; ^3^ Department of Orthopaedic Surgery, China-Japan Friendship Hospital, Beijing, China

**Keywords:** atlantoaxial dislocation (AAD), cervical spondylotic myelopathy (CSM), hybrid surgery (HS), craniovertebral fusion, laminoplasty

## Abstract

**Background:**

To introduce a hybrid surgery of posterior craniovertebral fusion plus subaxial laminoplasty for atlantoaxial dislocation (AAD) coexisting with multilevel cervical spondylotic myelopathy (CSM).

**Methods:**

A retrospective study was performed by reviewing data from 23 patients with the coexistence of AAD and CSM who underwent the hybrid technique (*n* = 23). Clinical outcomes, including visual analogue scale (VAS), Japanese Orthopaedic Association (JOA), and neck disability index (NDI) score, and radiological cervical alignment parameters including C0–2 and C2–7 Cobb angle and range of motion (ROM) were analyzed. The operation time, blood loss, surgical levels, and complications were recorded.

**Results:**

The included patients were followed up with an average of 20.91 months (range, 12–36 months). Clinical outcomes including JOA, NDI, and VAS scores were significantly improved at different postoperative follow-up points. C0–2 Cobb angle, C2–7 Cobb angle, and ROM showed a stable tendency after 1-year follow-up. No major perioperative complications occurred.

**Conclusion:**

This study underlined the importance of pathologic condition of AAD coexisting with CSM and presented a novel hybrid approach of posterior craniovertebral fusion plus subaxial laminoplasty. This hybrid surgery was effective in achieving the desired clinical outcomes and better maintaining cervical alignment, proving its value and safety as an alternative technique.

## 1. Introduction

Atlantoaxial dislocation (AAD) was a potentially fatal pathological condition derived from various etiologies (1). In recent years, sagittal balance of the cervical spine has aroused increasing attention (2). Indeed, the upper and lower cervical spine have a reciprocal relationship in natural or pathological conditions (3–5). Under the circumstances, the coexistence of AAD and cervical spondylotic myelopathy (CSM) may be an underestimated disorder. It was discussed extensively that prompt reduction and fixation were the keys to treatment for AAD. The conventional surgical method for combined AAD with CSM was the posterior long segmental decompression and fusion. However, it was reported to have higher postoperative VAS, complication rate, and surgical trauma owing to more concentrated stress and increased compensatory motion (6).

The hybrid technique was the combination of fusion plus non-fusion or static plus dynamic technique that practically preserved the segmental range of motion (ROM) and reduced the instrument-related risk and adjacent segment degeneration rate. Several biomechanical and clinical studies investigated the potential benefits of the hybrid methods over fusion alone for CSM (7, 8). In a case series, Li et al. performed occipital–cervical fusion combined with laminoplasty or single laminoplasty extending to the C2 level for upper cervical canal stenosis and ossification of the posterior longitudinal ligament (OPLL), in which the pathological conditions and radiological manifestations were discussed. Nevertheless, data regarding hybrid technique involving the upper cervical spine remain scarce. Here, we aimed to describe our case series utilizing a hybrid method of posterior craniovertebral fusion plus multilevel subaxial laminoplasty for AAD coexisting with CSM.

## 2. Materials and methods

### 2.1. Patients

This retrospective analysis focused on patients with the coexistence of AAD and CSM who underwent posterior craniovertebral fusion plus multilevel subaxial laminoplasty from June 2014 to December 2021 at our hospital. The present study was approved by the local ethics committee. Informed consent was obtained and all patient data were anonymous and kept confidential.

The inclusion criteria were patients with the coexistence of AAD and CSM confirmed by conventional radiographs, CT and MR imaging, and patients who had been followed up for at least 12 months after the surgery. The exclusion criteria were patients with infection, tuberculosis, tumor, or unable to undergo surgery owing to poor cardiopulmonary function or hepatic or renal failure; patients with previous spinal surgery; and patients with cervical kyphosis alignment.

### 2.2. Operative procedures

#### 2.2.1. Preoperative preparation

Radiological evaluations included plain x-ray films, CT with three-dimensional reconstruction, and MRI. All patients underwent 2 weeks of skull traction upon admission starting with 3 kg and gradually increasing to 6–10 kg. The anterior release was decided owing to unsatisfied reduction verified by dynamic flexion and lateral extension as described in our previous research study (9), or else patients were considered to undergo a single posterior approach operation. The same chief spinal surgeon performed all surgeries in the upper cervical region with more than 20 years of surgical experience.

#### 2.2.2. Surgical technique

All patients underwent general anesthesia in a supine position. After routine sterilization, the autogenous iliac bone was harvested and changed to the prone position. A posterior midline incision was made extending from the occipital protuberance to the target spinal process. Two different surgical procedures were applied respectively, according to the state of dislocation and bone morphological condition. If occipitocervical instability was caused by deformities of the C1 vertebrae or basilar invagination, occipitocervical fusion was performed. In other cases, atlantoaxial fusion was routinely utilized. The paraspinal muscles and tissue was dissected carefully to reveal the occipital bone, the vertebral body of C2, and the bilateral lamina of the subaxial cervical spine. After penetrating the bone cortex at the midpoint of the C2 lateral mass with ultrasonic osteotomy, the pedicle or lateral mass screws were placed on both sides of the C2 vertebra. Lateral mass screws were used for the lower cervical spine with a depth-limited drilling each time and subsequently a probe was used to explore all the walls of the trajectory. An appropriate occipital plate was selected and fixed on the surface of the occipital protuberance. The posterior arch of the atlas was carefully dissected to approximately 15 mm from the midline, and the venous plexus and vertebral artery were carefully exposed along with the posteroinferior border of the posterior arch. The bone was then grooved on both sides of the posterior arch with an ultrasonic osteotome, and part of the posterior arch was uncovered to relieve compression of the dural sac. When C1–C2 pedicle screws can be fixed, the entry point at the posterior arch of the atlas was carefully dissected to approximately 18–22 mm from the midline. The vertebral artery and the vein plexus between C1 and C2 superiorly and inferiorly to the C1 posterior arch were meticulously exposed for screw placement. The pilot hole was created by a high-speed burr and deepened with a depth-limited drill. Subsequently, a probe was used to explore all the walls of the trajectory in case of penetration and 3.5 mm screws were placed. Preflexed and connected with two appropriate titanium rods were preflexed and fixed to the screws bilaterally. When the rods were fixed, the pull-out strength could reconstruct the alignment. After decortication of the laminae and facet joint, an iliac crest graft was modified to implant on the posterior rim of the occipital and C1–2. Then, additional multilevel subaxial laminoplasty was scheduled. The open side was determined by the side with the greater degree of symptoms. After titanium rods were fixed in the process described above, the outer cortex of the lamina at the hinge side of the target vertebrae and the full-thickness cortex of the lamina at the opening side at 1–2 cm from the spinous process were removed. For the segment undergoing simultaneous laminoplasty and screw fixation, the entry point was slightly lateral to the outside or the gutter position was appropriate to the inside, to avoid impeding grooving and lifting of opening-side lamina by screws. The lamina door was lifted with the hinge side as fulcrum gently and a periosteal detacher was assisted in achieving the opening angle of 30–40°. A 1 mm rongeur was used to repair the marginal bone and remove part of the ligament flavum. A titanium plate of suitable size was placed between the lamina and the lateral mass at the opening side, and each lamina was fixed with four screws.

#### 2.2.3. Postoperative management

After this period, all patients were routinely treated with anti-inflammation, detumescence, and nutritional nerve therapy. The drainage tube was removed when there was no cerebrospinal fluid leakage or the drainage fluid was less than 50 ml per 24 h. The cervical brace was maintained for at least 3 months.

### 2.3. Clinical evaluation

VAS (10 scores) was used to compare the pain intensity, and JOA (17 scores) was assessed to evaluate the improvement of neurological function. NDI (50 scores) was used to determine the health status. We defined bony fusion as no absorb or translucent line around the graft, no instrument failure, and no movement under a dynamic radiograph.

### 2.4. Radiological assessment

**Figure 1:**
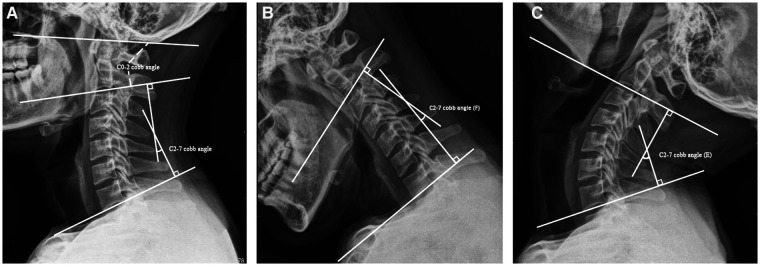
The definition of radiological assessment. (**A**) The definition of C0–2 and C2–7 Cobb angles. (**B,C**) The cervical ROM was defined as the difference between the C2–7 Cobb angle of flexion–extension lateral radiographs. ROM, range of motion.

X-ray, 3D CT, and MRI examinations were performed before the operation and during the follow-up to observe the instruments, graft, reduction, and stability. Data measurements were performed three times by the first and second authors independently through the Picture Archiving and Communication Systems (PACS, Carestream Health, Inc., Shanghai, China) in our department, and the mean value was used for analysis. The C0–2 and C2–7 Cobb angles were measured on lateral x-rays. The C0–2 Cobb angle was determined by the McGregor line and a line extending from the inferior aspect of the C2 vertebral endplate. The C2–7 Cobb angle was measured between a line extending from the inferior aspect of the C2 vertebral endplate and the inferior line of the C7 vertebral endplate (Figure 1A). The ROM was the difference between the C2–7 Cobb angle of flexion–extension lateral radiographs (Figures 1B,C). The patients were encouraged to wear a firm cervical collar for 3 months; thus, we did not measure the cervical range of motion 1 month after surgery.

### 2.5. Statistical analysis

All statistical analyses were performed using SPSS 26.0 software (SPSS, Inc., Chicago, IL, United States), and data were expressed as mean and SD. The statistical differences among the baseline characteristic data before surgery were determined by paired-sample *t*-tests. Numerical variables at different follow-up times were assessed by repeated measures ANOVA. The Mann–Whitney *U* test or Fisher's exact tests were utilized for nonparametric comparisons. *P *< 0.05 was considered to have statistical significance.

## 3. Results

### 3.1. Baseline data

**Figure 2:**
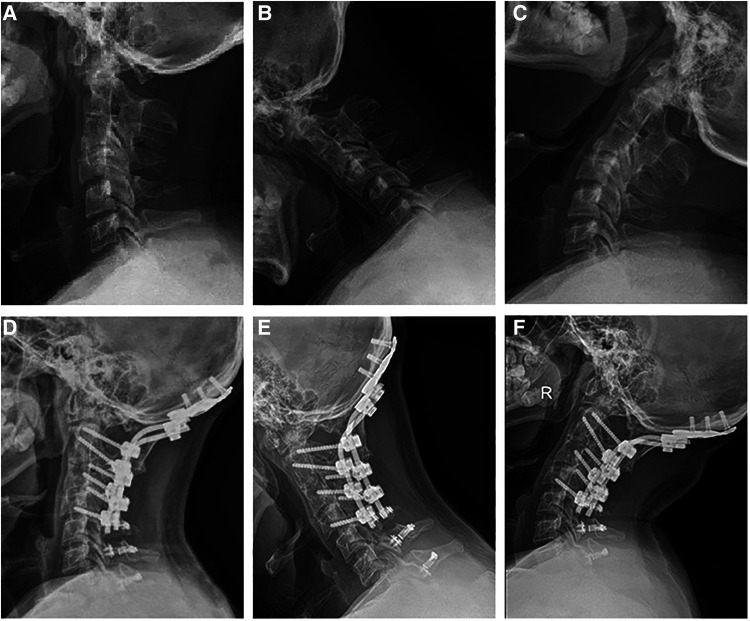
A 41-year-old male underwent a hybrid surgery for combined posterior occipitocervical fusion and subaxial laminoplasty (C0–7). (**A–C**) Preoperative lateral radiographs showed that the C0–2 Cobb angle was 15.5°, C2–7 Cobb angle was 24.6°, and ROM was 54.3°. (**D–F**) Last follow-up lateral radiographs showed that the C0–2 Cobb angle was 17.2°, C2–7 Cobb angle was 24.2°, and ROM was 36.15°. ROM, range of motion.

The general characteristic data of the included patients are shown in Table 1. A total of 23 patients were analyzed retrospectively in this study. All cases were followed for 20.91 ± 6.73 months. The illustrative case of the hybrid technique is shown in Figure 2.

**Table 1:** Baseline characteristic data of included patients.

Variable	Value (*n* = 23)
Demographics	
Age (year)	44.91 ± 10.89
Gender, M/F	13/10
Mean follow-up (months)	20.91 ± 6.73
Surgery information	
Surgical levels	5.35 ± 0.96
Operation duration (min)	314.35 ± 74.79
Blood loss (ml)	422.39 ± 156.68
Preoperative clinical parameters	
JOA	8.22 ± 2.43
VAS	7.09 ± 0.97
NDI	21.3 ± 9.13
Preoperative radiographic parameters	
C0–2 Cobb angle (°)	11.17 ± 6.94
C2–7 Cobb angle (°)	13.22 ± 3.57
ROM (°)	35.87 ± 9.02

ROM, range of motion.

### 3.2. Clinical outcomes

**Figure 3:**
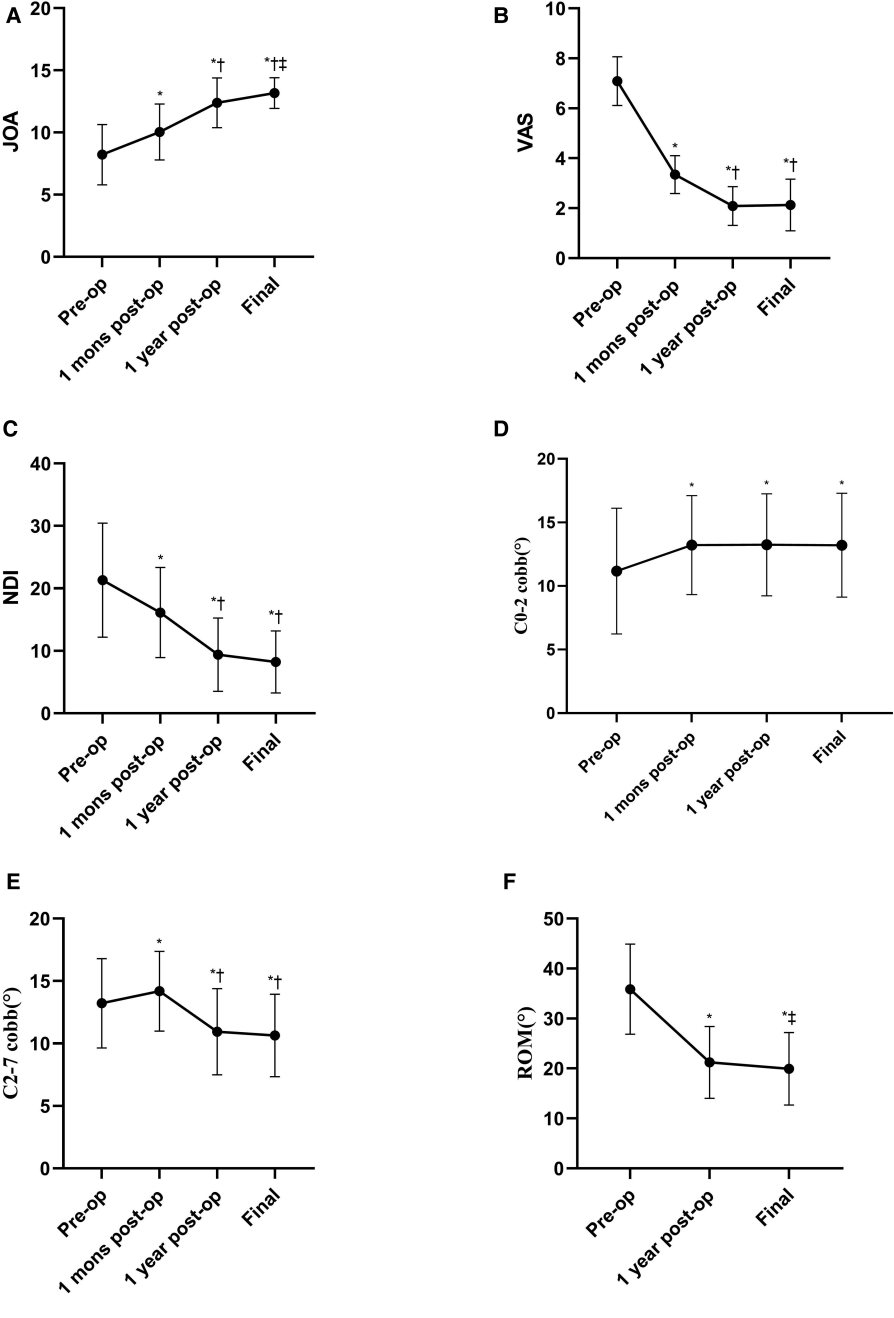
Radiological outcomes and JOA, VAS and NDI scores at 1 month, 1 year and the final follow-up after surgery. (**A**–**C**) *P < 0.05 compared with the preoperative JOA, VAS and NDI scores. †*P* < 0.05 compared with the 1 month postoperatively. ‡*P* < 0.05 compared with the 1 year postoperative JOA. (**D**–**F**) *P < 0.05 compared with the preoperative C0-2, C2-7 cobb and ROM. †*P* < 0.05 compared with the 1 month postoperative C2-7 cobb. ‡P < 0.05 compared with the 1 year postoperative ROM.

As shown in Figure 3, there was a significant improvement in JOA, NDI, and VAS scores postoperatively. Specifically, the JOA score improved from 8.22 ± 2.43 preoperatively to 10.04 ± 2.26 at 1-month postoperative follow-up, 12.39 ± 1.99 at 1 year after the operation, and 13.39 ± 1.28 at the final follow-up (*F* = 120.34, *P* < 0.001). The VAS score improved from 7.09 ± 0.97 preoperatively to 3.35 ± 0.76 at 1-month postoperative follow-up, 2.09 ± 0.78 1 year after the operation, and 2.13 ± 1.03 at the final follow-up (*F* = 250.27, *P* < 0.001). The NDI score improved from 21.3 ± 9.13 preoperatively to 16.13 ± 7.21 at 1-month postoperative follow-up, 9.39 ± 5.87 1 year after the operation, and 8.22 ± 4.98 at the final follow-up (*F* = 91.74, *P* < 0.001).

No major perioperative complications occurred, such as cerebrospinal fluid leakage, instrumentation failure, neurological deterioration, or vascular injury. However, a superficial surgical site infection was observed in one patient, which was healed after intermittent debridement and antibiotics. In addition, C5 palsy was observed in two patients, which was alleviated by conservative treatments.

### 3.3. Radiological outcomes

Based on the conventional radiographs, the C0–2 Cobb angle was preserved 1 month after surgery (13.22 ± 3.89°), 1 year after surgery (13.24 ± 4.01°, *P* > 0.05), and at the final follow-up (13.21 ± 4.08°), with statistical difference compared to preoperative (11.17 ± 6.94°, *F* = 12.03, *P* < 0.05). C2–7 Cobb angle changed from 13.22 ± 3.57° preoperatively to 14.19 ± 3.2° at 1-month postoperative follow-up, 10.94 ± 3.45° 1 year after the operation, and 10.65 ± 3.3° at the final follow-up (*F* = 137.04, *P* < 0.001). As to cervical ROM, although there was a statistical reduction compared to preoperative (35.87 ± 9.02°), a tendency toward a stable ROM existed at 1 year after the operation (21.22 ± 7.18°) and at the final follow-up (19.94 ± 7.24°, *F* = 313.55, *P* < 0.001) (Table 2).

**Table 2:** Clinical and radiological outcomes before and after surgery.

Variable	Pre	1 m post	1 year post	Final	*F* value	*P* value
JOA	8.22 ± 2.43	10.04 ± 2.26	12.39 ± 1.99	13.39 ± 1.28	120.34	<0.001
VAS	7.09 ± 0.97	3.35 ± 0.76	2.09 ± 0.78	2.13 ± 1.03	250.27	<0.001
NDI	21.3 ± 9.13	16.13 ± 7.21	9.39 ± 5.87	8.22 ± 4.98	91.74	<0.001
C0–2 Cobb angle (°)	11.17 ± 6.94	13.22 ± 3.89	13.24 ± 4.01	13.21 ± 4.08	12.03	<0.05
C2–7 Cobb angle (°)	13.22 ± 3.57	14.19 ± 3.2	10.94 ± 3.45	10.65 ± 3.3	137.04	<0.001
ROM (°)	35.87 ± 9.02	—	21.22 ± 7.18	19.94 ± 7.24	313.55	<0.001

ROM, range of motion.

## 4. Discussion

The coexistence of AAD and CSM may be an underestimated disorder. The upper and lower cervical spine have a reciprocal relationship in natural or pathological conditions. Limited subaxial cervical spine mobility caused by various pathological conditions in CSM may be the significant reason for upper cervical spine hypermobility, and AAD is also frequently associated with subaxial multilevel cervical instability in degenerative diseases attributable to segmental joint instability (10). Gong et al. (4) indicated that atlanto-occipital joint flexion stiffness was closely correlated with a high risk for the occurrence of cervical spondylosis. Kim et al. (11) proved that a small ROM at C0–1 combined subaxial laminoplasty is a risk factor for subaxial kyphotic change. The cervical canal further decreases owing to the presence of craniovertebral lesions (12). In addition, changes in the balance of the lower cervical spine indubitably generated secondary changes in the upper cervical spine (13). Hence, consideration should be given to achieving an overall balanced cervical alignment by preserving the physiological curvature and reasonable range of motion to the greatest extent.

As a potentially fatal pathological condition, it is widely accepted that prompt reduction and fixation are crucial for the management of AAD (14). The posterior pedicle screw fixation through the posterior arch at the C1 level has become a recognized surgical method due to the clinical advantages including desired pull-out resistance, less bleeding, and lower risk of postoperative complications, while lateral mass fixation under the posterior arch is an alternative approach if the posterior arch was too thin (15, 16). Anterior release, C1 laminectomy, and craniovertebral fusion are performed if necessary. In recent years, upper cervical laminoplasty is proposed, which achieves promising clinical outcomes allowing for the most drifting of the spinal cord; nevertheless, it is contraindicative to atlantoaxial instability (5, 17). Conventional long segmental posterior decompression and fusion (PDF) for the coexistence of AAD and CSM has been widely utilized with concomitant fusion-related complications. Although it is broader recommended for preserving segmental stability, sufficient cervical lordotic curvature, and global cervical balance after upper cervical rigid fixation, it may encounter higher postoperative VAS, complication rate, and surgical trauma owing to more concentrated stress and increased compensatory motion.

In this study, hybrid surgery of posterior craniovertebral fusion plus multilevel subaxial laminoplasty was performed, allowing for preserving comparable sagittal alignment and more significant cervical motion. Our results showed improved clinical parameters (JOA, VAS, and NDI scores) at the final follow-up similar to a previous study (12), confirming the validity of the hybrid method for the coexistence of AAD and CSM. Moreover, there was a stable tendency in C0–2 Cobb angle, C2–7 Cobb angle, and ROM. In general, cervical laminoplasty is a non-fusion or dynamic technique implemented as a direct decompression of posterior compressive lesions and an indirect decompression of the anterior compressive elements. It is commonly recommended for patients with CSM, particularly with neutral or lordotic cervical sagittal alignment without axial neck pain and substantial instability (18). Given that the lack of posterior vertebral structure due to laminoplasty had a significant impact on cervical sagittal balance, which may further lead to subsequent clinical outcomes deterioration (19), patients with neutral or lordotic cervical curvature were strictly selected. Theoretically, with an additional laminoplasty, prolonged or skip decompression could be performed within one operation regardless of mild to severe degrees of spinal stenosis, which avoided concerns about excessively prolonged fusion for mildly or moderately stenotic levels and consequently increasing the risk of complications and morbidity. In addition, the one-stage posterior approach may avert more intraoperative procedures and postoperative complications than the anteroposterior approach.

This study could provide baseline insights into various clinical studies on the management of patients with the coexistence of AAD and CSM. In 2009, Shin et al. (20) first proposed the concept of cervical hybrid surgery based on the respective indications and postoperative complications of anterior cervical discectomy and fusion (ACDF) and cervical artificial disc replacement (CADR). Of note, the hybrid method was the combination of fusion plus non-fusion or static plus dynamic technique, compensating for the deficiency of single CADR or ACDF in theory (21). Indeed, the hybrid methods was also an attempt to perform specific procedures for different segments given that not all degenerative levels conform to the identical indication of a particular approach in CSM (22). In recent years, it has been validated that hybrid surgery practically preserves the segmental range of motion compared to the fusion technique and reduces the instrument-related risk and adjacent segment degeneration rate (23). The merit of the anterior approach was the direct relief of the anterior compression without manipulating the spinal cord. Nevertheless, due to the accelerated risk of reconstruction failure occurring after the anterior multiple-level procedure, the posterior approach was preferred for multisegment CSM involving three or more levels (24, 25). To date, the analysis regarding posterior cervical hybrid surgery is of scarcity, let alone a combination of craniovertebral fusion and laminoplasty. Motosuneya et al. (26) described one successful case that underwent occipital–cervical fusion utilizing a hook-and-rod system for AAD, and double-door laminoplasty at C3–7 levels for CSM. Li et al. (12) delivered a case series study concerning patients with simultaneous upper cervical canal stenosis and OPLL, part of which underwent occipital–cervical or atlantoaxial fusion combined with laminoplasty for congenital deformities, AAD or OPLL. The pathological conditions and radiological manifestations were discussed in particular to select the appropriate surgical strategy. Based on our clinical experience, internal fixation was performed prior to groove as to the segments with simultaneous laminoplasty and screw placement. This sequential procedure provided advantages as follows: (1) The complete anatomical structure and entry point position were retained, facilitating screw placement; (2) This method obviates the risk of damaging the exposed spinal cord due to the possible inappropriate management through screw placement; (3) The titanium rods were preflexed and fixed to rectify the cervical spine’s physiological lordosis, which was beneficial in restoring postoperative curvature. In addition, the entry point was slightly lateral to the outside of the gutter position appropriate to the inside, to avoid impeding grooving and lifting by screws. Whether laminectomy and fusion or laminoplasty were performed relied on vertebral stability and the severity of spinal cord compression. Total laminectomy and fusion for the segment were recommended in patients with severe developmental spinal stenosis or ossification of the ligamentum flavum with an unstable state.

There were several limitations in the present study. Chief among was the small sample size and relatively short-term follow-up. This was mainly due to the paucity of patients. Another limitation was retrospective in nature. Further large control studies are warranted and may have more standardized outcome measures.

## 5. Conclusion

The pathological condition of AAD coexisted with CSM should be properly considered. We found that the hybrid surgery of posterior craniovertebral fusion plus multilevel subaxial laminoplasty may achieve the desired clinical outcomes with improved postoperative clinical parameters regarding JOA, VAS, and NDI scores. Moreover, there was a stable tendency in C0–2 Cobb angle, C2–7 Cobb angle, and ROM, which was effective in maintaining cervical alignment, proving its value and safety as an alternative technique.

## Data Availability

The raw data supporting the conclusions of this article will be made available by the authors, without undue reservation.
